# Application of the Laser Linear Distance-Speed-Acceleration Measurement System and Sport Kinematic Analysis Software

**DOI:** 10.3390/s22155876

**Published:** 2022-08-05

**Authors:** Stanko Štuhec, Peter Planjšek, Mariusz Ptak, Milan Čoh, Krzysztof Mackala

**Affiliations:** 1 Faculty of Sport, University of Ljubljana, Gortanova 22, 1000 Ljubljana, Slovenia; 2 Ljubljana School of Business, Management and Informatics, Tržaška cesta 42, 1000 Ljubljana, Slovenia; 3 Faculty of Mechanical Engineering, Wroclaw University of Science and Technology, Lukasiewicza 7/9, 50-371 Wrocław, Poland; 4 Faculty of Physical Education and Sport, Wroclaw University of Health and Sport Science, Paderewskiego 35, 51-612 Wrocław, Poland

**Keywords:** speed, sprints, laser system, step kinematics, run measurements, biomechanics

## Abstract

The industrial development of technology, with appropriate adaptation, enables us to discover possibilities in sport training control. Therefore, we have developed a new approach to linear running analysis. This study aims to determine the measurement possibilities using an LDM301A laser system in obtaining basic kinematic parameters. The second goal is the application of specialized computer programs based on appropriate algorithms to calculate a vast number of variables that can be used to adjust the training and the rivalry. It is a non-invasive, non-contact measurement method. We can also determine the influence of both subjective and objective external factors. In this way, we can also conduct training with real-time scientific feedback. This method is easy to use and requires very little time to set up and use. The efficiency and running economy can be calculated with various time, speed, acceleration, and length indexes. Calculating the symmetries between the left and right leg in velocity, stride lengths, support phase times, flight phase times, and step frequency are possible. Using the laser measurement method and detailed kinematic analysis may constitute a new chapter in measuring speed. However, it still has to compete with classic photocell measurement methods. This is mainly due to their high frequency of measurement used, despite some reservations about the scale of measurement errors.

## 1. Introduction

Training control based on new technologies and technological–methodological solutions are essential in sport. These procedures aim to determine the relevant and objective parameters of the athlete’s current speed preparation. Without data on biomotor, morphological, physiological, biochemical, psychological, and sociological characteristics, it is unmanageable to plan, program, and model a modern training process [1,2,3,4,5]. Based on the measured variables, we can choose the most effective methods and means for planning training and, thereby, improving sport results.

The athlete’s motor skill development and special conditioning preparation interact in the sport training process [6,7]. This relationship is dynamic and always different depending on the phases of the training process and the biological development of the athlete [1,7,8]. Given that automated stereotypes and the level of motor abilities are changing, the training process must be monitored, controlled, and finally, corrected. The speed of the sprint changes in the individual phases of the run, thus each stage deserves special treatment, both in terms of training control and training itself [9]. Notably, the sprint is considered as running up to 100 m. The advantage of our new training control method is the help of a laser measurement, which is very useful because, in many sport disciplines, the ability to accelerate well from a static position and quickly develop the highest running speed is the key to improving performance in both teams and individual sports. Running velocity is the composition of stride frequency and stride length; in studies where the same subjects ran at different speeds, both stride rate and stride length are highly correlated with increasing running speed [1,10].

Current information, biocybernetic, and visual technologies solve the most demanding movement problems in the diagnostics of the sport training process [11,12,13,14]. The conceptualized modern measurement allows for an objective analysis of the movement structures, the selection, and the application of the most suitable training control methods for individual modelling of athletic training [15]. Locomotor speed is undoubtedly one essential biomotor ability that improves sport performance. It occurs in various sport disciplines such as running, sprinting, or jumping. In recent decades, the main focus has been on diagnostic capabilities that allow monitoring changes in the kinematic values of sprint parameters. This applies to both the competition and the monitoring of the training process. It is only possible due to the application of appropriate measurement methods (fully automatic timing systems, photocells, Optojump System, video-recording via high-speed camera, global positioning systems—GPS, or laser system) and proper software, enabling an insightful analysis. This last methodology was used in the most critical athletic competitions in the world (World Championships, Olympic Games). The LDM (laser distance measurement) device appears to be an entirely new chapter in developing speed training control methods.

The most desirable information about a moving athlete can be obtained from the description of the linear speed—the movement of SG (center of gravity). Such a reference is essential when the direction of the human body with all segments is studied as a maximal speed of a single point [16]. With limitations, an LDM can also be used to set realistic conditions for acceleration—deceleration drills [17]. Results in research indicate that a low-cost and accessible laser system can be used to accurately determine walking and running speed [18]. Therefore, the aims of this study are twofold. First, to present the usefulness of data acquisition from the LDM 301A (ASTECH GmbH, Rostock, Germany) device during the maximum sprint. The second goal was to determine the analytical capabilities of a new software, and consequently, to evaluate the usefulness of this software for analysis of speed kinematic profiles.

## 2. Materials and Methods

### 2.1. Participants

The measurement of the 100 m sprint with the LDM 301A (ASTECH GmbH, Rostock, Germany) system involved one national-level Slovenian sprinter (age 22.4 years, body height 177.6 cm, and body weight 74.9 kg; best result 10.39 s/100 m). Before the experiment, the subject had six years of active training and competition in sprinting (60, 100, and 200 m). The laser measurement occurred at the beginning of the competition period, at the Faculty of the Sport, University of Ljubljana stadium. It was a sunny day, and the wind was +1.2 m/s. Before the study, approval by the Human Ethics Committee of the University of Ljubljana was obtained for this experiment. The participant was notified about the risks associated with participating in this experiment, the purpose of the investigation, and the measuring procedures. He signed an informed consent document before any testing.

### 2.2. Description of the Laser Distance Measurement Device

**Figure 1:**
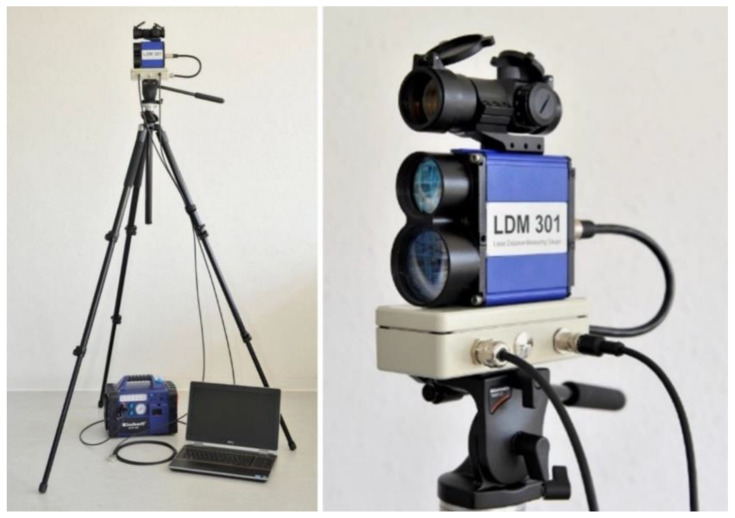
Laser distance measurement system setup (**left**) and LDM301 device with pivot laser point and base station (**right**).

A laser distance measurement device is completely non-invasive, which in practice means that an athlete can run in competition conditions without any sensors on the body. The LDM301A with an invisible beam (Figure 1) is a Class 1 laser device based on the norm IEC 60825-1:2003. The connection to a computer was established via a particular base station using the RS232 to USB port converter. The pilot laser with a visible redpoint is a Class 2 laser device based on the norm IEC 60825-1:2007.

**Figure 2:**
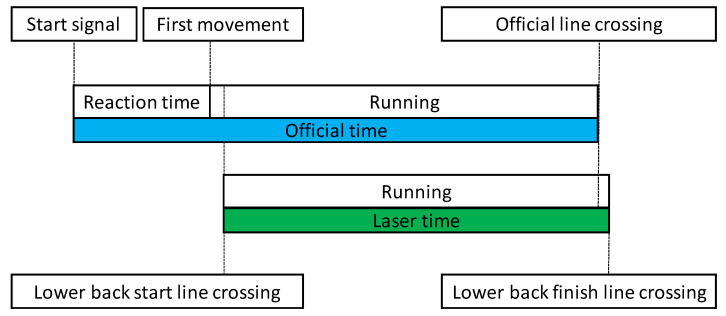
A schematic representation of the official measurement compared to a laser distance measurement used in the study.

The average angle of the laser beam spread to 1.7 mrad. The divergence of the laser was 1.7 mrad × 0.08 mrad (rectangle). The receiver divergence was 2.9 mrad (circle). The size and shape of the laser beam on the reflecting surface increased with distance. From ten to one hundred meters, the surface of the laser beam increased by a factor of 12.9 (from 1548 mm^2^ to 19,900 mm^2^). The measurement precision of the device was ±20 mm in a mode measurement frequency of 2 kHz and measurement value output of 100 Hz. The LDM tool created a text file containing the measurement data (first row, time; second row, distance). A schematic representation of the official measurement compared to a laser distance measurement used in the study is depicted in Figure 2. 

### 2.3. Measurement Method

**Figure 3:**
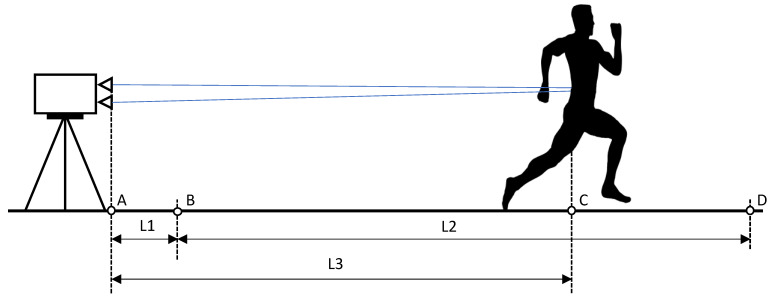
Diagram showing a sprint measurement protocol using a laser distance measurement. Legend: A—laser position, B—starting line, C—distance to the lower back of the runner, D—finish line, L1—calibration distance, L2—measuring distance, L3—the actual measuring distance of lower back to the laser measurement (L2 is calculated as the difference between L3 and L1).

Before starting the measurement, we needed to calibrate the track with a particular calibrating device. The calibration was performed by placing a rectangular bar (height 1.5 m, width 0.03 m, and depth 0.03 m) in an exact vertical position on the starting line and measuring the distance to the bar with the laser. The laser must be in a precise horizontal position and at the exact height of the lumbar spine of the measured person (L1 in Figure 3). This distance represents the basis for the measurement. When the sprinter passed the measured calibration distance, the sprinter entered the measurement zone (L2 in Figure 3). The measurement lasted as long as the sprinter’s lower back was in the measurement zone (L2).

Due to various factors, some errors were made in the raw measurement. The most common errors occurred due to a loss of laser contact with the lower back due to the movement of the sprinter left to right, interruptions of the laser beam by hand, low-reflection material of the shirt, and other interruptions to the laser signal.

### 2.4. Data Processing

For raw distance-time data capture, we used the original software LDMTool from ASTECH. A simple user interface displayed all available parameters of the currently connected sensor and all measured values, as well as the state of the different output signals. There were four main groups of direct communication: device parameters, device data, a graphical display of a distance-time diagram, and a log window.

The raw displacement data obtained with the LDM device were captured with a frequency of 100 Hz. From the change in displacement, the speed sprint (vh) was calculated in every hundredth of a second of running. The curve was smoothed with a moving average filter over a 0.1 s interval (n = 10, smoothing frequency m = 10) to eliminate any within-step velocity fluctuations. The polynomial startpoint was identified from where the raw displacement values increased and remained more significant than 2 SD above the mean noisy pre-start signal level. The endpoint was 50 data points after the displacement exceeded 60 m. 

### 2.5. Software for Kinematic Laser Distance Linear Running Analysis

At the University of Ljubljana, Faculty of Sports, Institute of Sports, we developed an entirely new approach to kinematic measurements of linear sprint running in the Biomechanical Laboratory. The development of laser meters, which allows us to perform non-contact measurements of horizontal position, speed, and acceleration, combined with our program, has enabled us to quickly and accurately diagnose running techniques and tactics. With entirely new algorithms and the already mentioned essential variables, we additionally calculate the support time, flight time, step time, and frequency within individual steps. Advanced functions allow us to calculate the symmetries between the left and right foot for each step and step phase. The horizontal running speed of the sprinter fluctuates from the maximum speed at the last contact of the push-off leg with the ground, and then decreases due to air resistance and the force of gravity in the flight phase until the opposite leg touches the ground, where it starts to increase again due to the action of the muscles in the push-off phase until the last contact time when maximum running speed is again reached on the opposite push-off leg. The time of the support phase is defined from the moment when the slope of the speed reduction curve changes and starts to increase again until the moment of maximum speed. The time of the left step lasts from the first contact of the left foot with the ground through the push-off time (the time until the last contact with the ground) and until the first contact of the right foot with the ground. The time of the right step lasts from the first contact of the right foot with the ground through the push-off time (the time until the last contact with the ground) and until the first contact of the left foot with the ground. The flight time of the left step lasts from the last contact of the left foot with the ground to the first contact of the right foot with the ground. The flight time of the right step lasts from the last contact of the right foot with the ground to the first contact of the left foot with the ground. When we have certain time phases of the left and right steps, we also calculate the step frequency, where we can define the beginning and end of the left or right step in different ways (the last contact of the left foot with the ground, through the flight phase from the left to the right foot and until the last contact of the right foot with the ground or from the first contact of the left foot with the ground to the last contact of the left foot with the ground, through the flight phase from the left to the right foot and until the first contact of the right foot with the ground). In the following, we calculated the new shape of the separate curve for path, velocity, and acceleration by prescribing the best match of the curve shape according to the wavelength, amplitude, and direction of inclination of the average section of each variable. The first derivative of path change over time is velocity, and the second derivative of it is acceleration.

The software was based on functionality divided into twelve modules: (1) data editing: data preview, data preparation, adding missing data, data cut, error data elimination, converting data to sprint format, and data save; (2) measurement description: place, date, wind, official time, reaction time, measured distance, name, birth date, country, height, weight, left leg length, right leg length, sport discipline, dominant hand, dominant leg, first leg on start, moving or static start, static high or low start, notes, calibration distance, and maximum speed tolerance; (3) data smoothing: the type and rate of the smoothing used for each variable; (4) data calculation: times, speed, acceleration, steps, zones, phases, sections, frequencies, symmetry index, efficiency index, effectiveness index, and performance index; (5) single analysis: analyze a single measurement; (6) databases: database organization; (7) multiple analysis: analyze and compare multiple measurements; (8) statistical: descriptive statistical data analysis; (9) artificial intelligence: with the use of machine learning and artificial intelligence, we developed a unique code to look for new ways of optimizing and improving the efficiency and effectiveness of running; (10) export: exported all raw and calculated data in txt, CSV, or xlsx data format; (11) report: graphical and numerical report (diagrams and tables); (12) print: print selected report options.

## 3. Results

**Figure 4:**
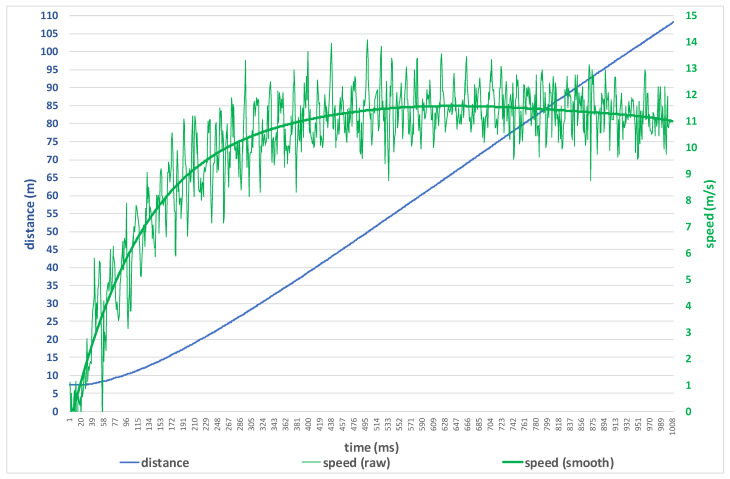
Measured raw distance-time data (blue line), calculated raw speed (thin green line), and averaged speed (bold green line).

Regarding the maximum running speed, both timing and distance data are essential. Based on this, we can adjust the training in real time so that the athlete can check on which section of the track he reached the maximum speed on each sprint. Therefore, raw data obtained during the measurement is a critical issue. It was first processed with the raw data editing module. The next phase is the data cut module. In this phase, we removed unwanted data before movement started and unwanted data after the lower back crossed the finish line. We recommend keeping data for one second before point B and after point D in Figure 3. The measured raw data and filter data are depicted in Figure 4. 

**Figure 5:**
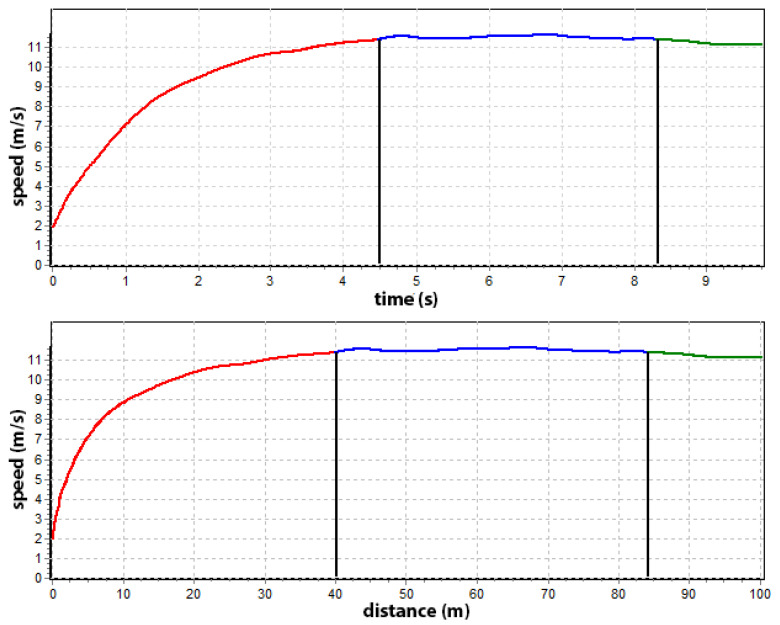
Sprinting phases: the diagram of speed in relation to time (above) and distance (below), acceleration phase (red line), maximum speed phase (blue line), and descending speed phase (green line).

Further, we can define the speed zones, which always appear in a 100 m race, no matter the performance level. It is possible to do this via smoothed speed data. The diagram shows the three primary phases of the 100 m run: red—ascending speed, blue—maintenance of the maximum speed, and green—descending speed (Figure 5). However, in this case, the maximum speed phase is defined by a tolerance of 2% from the maximum speed. An essential element of these two graphs is that the course of the variability of the maximum speed is presented in relation to the time of exercise and the change in distance. Both charts overlap and are the same. Maintaining a maximum speed is one of the most important indexes in sprint performance. 

**Figure 6:**
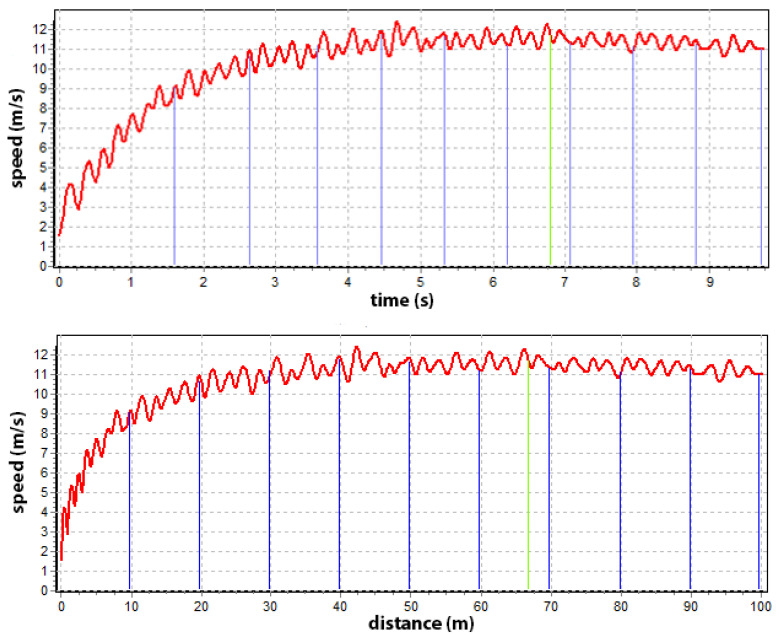
Speed-time (above) and speed-distance (below) with 10 m sections (blue lines) and point of maximum speed (green line).

This part of the analysis of the collected data is based on smaller, 10 m long segments of the 100 m distance, which allows studying the nature of the maximum speed in more detail. Such an analysis differs significantly from the overall approach because the course of individual running parameters is more distinct and is even due to smaller differences in the values of the discussed variables. Figure 6 shows how the sprinter’s speed increases proportionally concerning the average and maximum zone speeds. In this way, it can be found at what distance and when the sprinter reached a certain speed.

**Figure 7:**
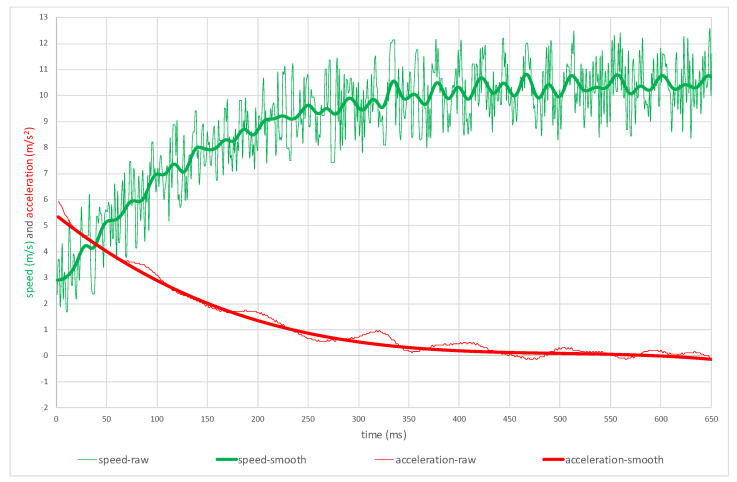
Speed and acceleration (raw and smoothed data).

In addition, our analysis of the speed of the object’s movement allows us to determine the type of movement, whether the runner is in an acceleration or deceleration phase. Consequently, the acceleration value expresses the speed of changing the position of a given object or the direction of its movement. More precisely, linear acceleration can be defined as the change of speed over time (1):

(1) $$ai=\frac{v}{t}=\frac{\left(vi+1\right)-\left(vi-1\right)}{\left(ti+1\right)-\left(ti-1\right)}$$

where: *a* = acceleration, *v* = speed, *t* = time, and *i* = calculation point. The calculated acceleration is one of the important variables used to determine sprint performance. We have to remember that the greater initial acceleration and longer positive acceleration define better performance. In Figure 7, we can observe the runner’s speed and acceleration in time. The peak velocity is calculated from the smoothed speed curve.

**Figure 8:**
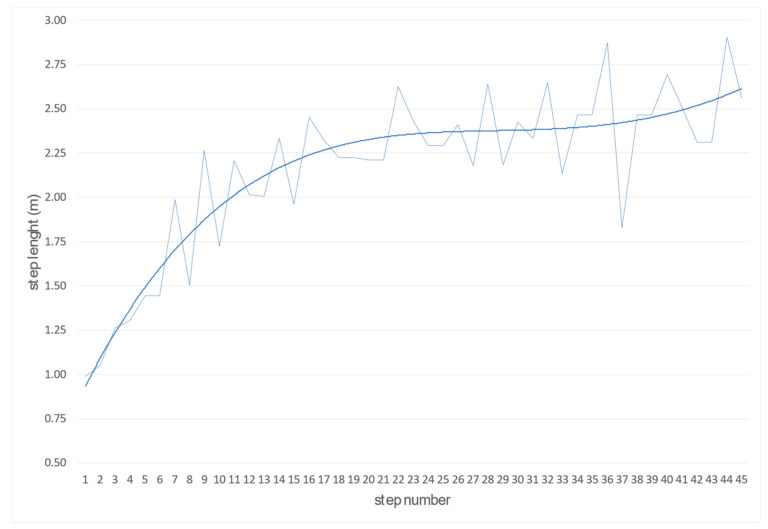
Function determining step length (thin line—raw and bold line—smooth length).

The primary criterion determining the effectiveness of a maximum speed is the length and frequency of steps and their mutual relations. High values of both these parameters have a decisive influence on the maximum speed value; they are also an indicator of the correct running technique. Using our program, we can accurately determine the length of each step. The module for automatically determining the length of the steps divides the entire run into individual steps based on the fluctuation of the speed of the run between the phase of the support phase and the flight phase. By smoothing, we eliminate the wrong calculated step length (Figure 8). 

**Figure 9:**
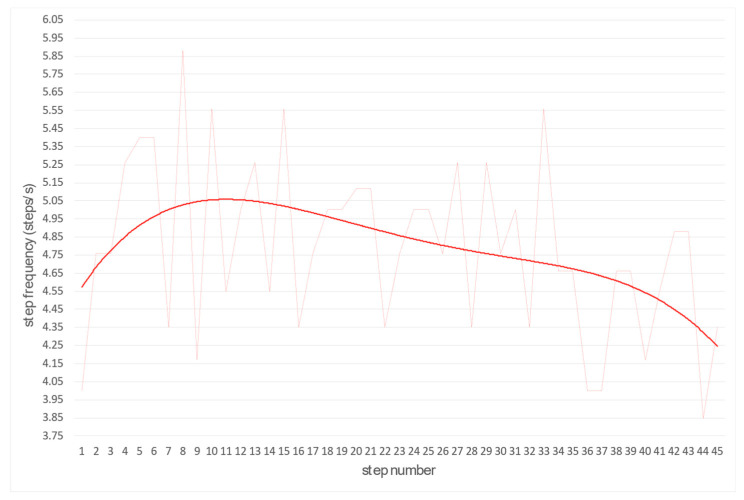
The function of determining step frequency (thin line—raw and bold line—smooth frequency).

**Figure 10:**
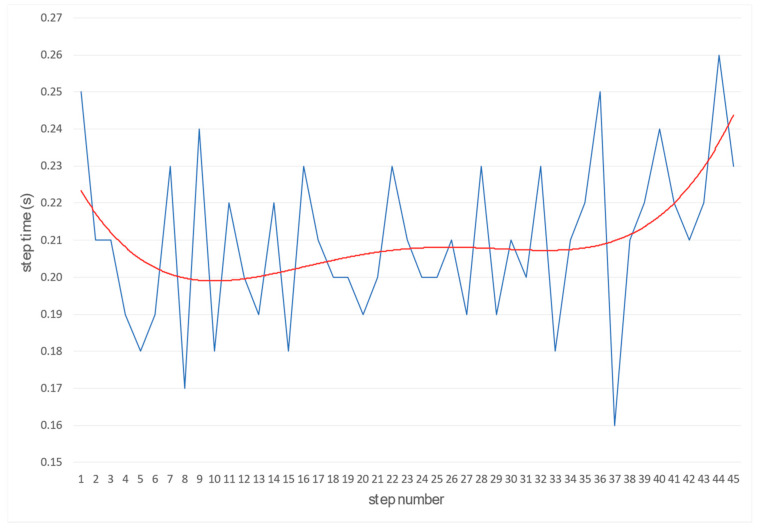
The function of determining step time from take-off (thin line—raw and bold line—smooth values).

By the same principle, the frequency of the steps and the time are also calculated (Figure 9 and Figure 10). Length and frequency of steps are the basis for optimizing and adapting these two variables to achieve a higher average running speed.

In the descriptive statistical data processing module, it is possible to calculate mean, median, mode, minimum, maximum, standard deviation, skewness, and kurtosis. We can also calculate the linear correlation between the selected variables. 

## 4. Discussion 

The aims of this study are twofold. First, assess the usefulness of a laser measuring system to record the time of a sprint run with maximum intensity. Secondly, propose an innovative computer software for the sprint kinematic analysis, based on the logarithm of the extraction of motion parameters, including information on the change in the course of the maximum speed in a straight line. The conclusion is that the time measurement made by using the LDM laser system is useful to provide data for a statistical analysis of the course of maximum speed changes during a sprint.

Exploring diagnostic problems related to sprint performance assessment, specifically the impact and magnitude of various external conditions, technologies, and monitoring, is one of the essential factors in the training process. In this case, the focus is on the diagnostic possibilities related to monitoring changes in the value of the linear running speed during competition and speed training. It seems to be a key element in improving sprint performance. 

The cinematographic method (Omega’s fully automatic timing system, Fribourg, Switzerland or Sony DCR-PC105E, Japan) is one of the primary methods of studying and evaluating the movement technique [11]. The main advantage of this method is the lack of direct interference in the athlete’s motor task performance, which allows for the full and free use of acquired technical skills. In recent years, several technologies have appeared to measure a run at maximum speed in a non-invasive way, both in a straight line and in curvature. There is an advanced technology that provides positional data with high spatiotemporal resolution [19]. The data can be collected with a radio-based position detection system (RedFIR, Fraunhofer Institute, Germany). The second option is to apply inertial measurement units to capture multidimensional accelerometers and gyroscope data to measure the kinematic parameters of a system. During the last decade, GPS with integrated accelerometers was extensively applied in various team sports to measure running velocity during training sessions and games [19,20,21,22]. One time-effective method for obtaining speed-time curves is applying a laser distance measurement (LDM) device [12,13]. The most recognizable and used at international athletic competitions (2008 World Championships and Olympic Games) is the LAVEG laser speed gun (LAVEG Sport, Jenoptik, Germany). The system is placed behind the starting line during the measurement, and the beam laser must be aimed (tracking the competitor) at his pelvis throughout the measurement [23]. The LAVEG system measures the positional information of an athlete at 100 Hz. Therefore, training with immediate feedback using a laser device can be of great help when we try to improve running performance.

An essential component of all these measurement methods is the validity and reliability of measurement [24,25]. Velocity data obtained from sprint trials were previously assessed but were limited, by comparison, to linear velocities at the hip over a distance. According to Haugen et al. [11], Omega’s fully automatic timing system demonstrated that the measurement method was valid to the instrument’s precision (±0.01 s), which is a very accurate value. The RedFIR (a radio-based position detection system) can provide precise in-field performance data based on reference systems [14,26]. Regardless of the level of validity and reliability, one crucial thing is that all these methodologies of time measurement systems allow obtaining the data that can be subjected to multidirectional analysis. It applies to velocity profiles—calculation of acceleration, instantaneous speed, split time, and parameters—that determine the length and frequency of the step.

The more precise this diagnostic, the better are the technology used, especially regarding the software for kinematic analysis. A proper kinematic analysis software of a sprint run should be based on a reliable acquisition of the primary structural properties of the movement, its characteristic quantities (numerical values), and the relations between them. Often, the identical movements of a sprinter’s lower limbs differ in many respects. This is mainly not due to different methods of recording a sprint run, but to the computer software applications used for detail analysis. A detailed description of the software operation can be found in the Materials and Methods section. However, this software aims to estimate the kinematic parameters (i.e., position, speed, acceleration, and possibly basic running step parameters) of the moving sprinter based on noisy measurements collected by the laser beam sensor. It is possible thanks to the use of a special tracking algorithm. It must consider the deterministic model of the dynamics of changes in the target’s position (e.g., model of acceleration or increasing speed). Such activity enables the estimation of the batch processing method (raw data from the running time) to obtain the target kinematic parameters of the sprint. Therefore, estimation of target kinematic parameters based on LDM measurements is not a template due to the linear nature of these measurements about the target kinematic parameters. Additionally, this software represents closed solutions and, thus, requires the repetition of numerical search algorithms to obtain accurate kinematic data. This makes this software highly effective.

The main task of training in a sprint should be to raise the athlete’s movement potential to the highest possible level to achieve maximum sport results [4,6]. This is mainly related to conducting comprehensive activities to achieve optimal running efficiency, maintaining the highest possible values of the maximum running speed over the entire distance [12]. The more significant the correlation between the length and frequency of running steps, the better.

The primary criterion determining the effectiveness of speed for a whole sprint run and the level of speed preparation of a competitor is the length and frequency of steps and their mutual relations [5,27]. High values of both these parameters have a decisive influence on the maximum speed value; they are also an indicator of the correct running technique. The research conducted by Luhtanen and Komi [10] and Mero and Komi [8] showed that the values of these parameters change with increasing speed, which has a linear course until the competitor develops a speed of about 7 m/s. As this value increases to about 9 m/s, the increment in the stride length is small, and the frequency increment is significant. This is even more evident when the competitors reach speeds of 11–12 m/s, which is only possible due to a substantial increase in pace [1]. In addition to the length of steps, the frequency of steps is the primary parameter that allows developing the maximum speed of the run, thus improving its efficiency. The more significant the correlation between the length and frequency of running steps, the better. However, this somewhat contradicts Deleclus’s manuscript [28], which found a linear relationship between the length of the running step and the speed developed, saying that there is no significant correlation between the frequency of steps and the speed. The author also believes that in short runs, the pace of the run reaches its maximum value at the beginning of the sprint (after a few steps) and does not change significantly.

On the other hand, the stride length increases almost the entire distance, which is an essential factor in developing maximum speed. The relationship between stride length and speed achieved in a 100 m run for men was determined based on linear regression analysis and was V = 0.79 + (3.89 Lk). The change in stride length can explain almost 85% of the variation in maximum running speed. In such an argument, it is essential to indicate whether the analysis considers only the distribution of the length of individual steps over the entire distance or the average value calculated for a 10 m section. The distribution curve shows a much greater variability (dispersion) between personal values for each competitor than the average value of the results obtained in the individual ten sections.

## 5. Conclusions

A runner’s distance, time, speed, and acceleration vary from step to step and can be divided into several phases and sections. Each of these phases has a particular influence on the final result. With LDM301A, the measured and calculated variables can be closely monitored at all stages, and each phase can be analyzed and optimized during the training process. Determining the right goals based on previous tests is crucial for the optimal planning of the athlete’s development. Once a coach has an insight into each phase, they can use the information to decide how to adapt the training process.

We will continue to develop the functions of the software in the future. One of the priorities is the variability of the take-off speed of the consistent steps and the take-off speed for the left and the right leg. Calculating acceleration within the steps can also give us information about the ratio of positive acceleration (propulsion phase—the second part of the support phase) to deceleration (flight phase and braking in the first part of the support phase). This way, a large amount of measurement data will give us a new insight into running optimization. We will also determine the new index between the maximum speed zone, step length, step frequency, and step time. Some of our data indicate a high probability of added value when planning sprint training with this approach.

## Data Availability

The data presented in this study are available on request from the corresponding author.
